# *Pseudomonas aeruginosa lasR* mutants resist phagocytosis and alter inflammatory cytokine production by cystic fibrosis macrophages

**DOI:** 10.1128/msphere.00702-25

**Published:** 2026-04-02

**Authors:** Daniel S. Aridgides, Diane L. Mellinger, Lorraine L. Gwilt, Anthony R. Correia, Carson E. Finger, Jay Goddard, Thomas H. Hampton, Dallas L. Mould, Deborah A. Hogan, Alix Ashare

**Affiliations:** 1 Section of Pulmonary and Critical Care Medicine, Dartmouth-Hitchcock Medical Center 22916, Lebanon, New Hampshire, USA; 2 Department of Microbiology and Immunology, Dartmouth Geisel School of Medicine 22916, Hanover, New Hampshire, USA; University of Nebraska Medical Center College of Medicine, Omaha, Nebraska, USA

**Keywords:** *Pseudomonas aeruginosa*, quorum sensing, host-pathogen interactions, phagocytosis, innate immunity, adaptive mutations

## Abstract

Cystic fibrosis (CF) is characterized by chronic muco-obstructive lung disease and infection. People with CF (pwCF) are often colonized with *Pseudomonas aeruginosa* for years to decades, allowing for evolutionary adaptation. In chronic *P. aeruginosa* lung isolates from pwCF, the quorum-sensing regulator LasR is frequently nonfunctional; however, the factors enabling *lasR* loss-of-function (LOF) mutant selection are incompletely understood. We hypothesized that LOF mutations in *lasR* could allow *P. aeruginosa* to resist the selective pressure of phagocytosis. We found that in multiple strain backgrounds, LasR LOF decreased phagocytosis by both model THP-1 and primary monocyte-derived macrophages, and *lasR* complementation increased phagocytosis in mutant strains. While exogenous administration of the quorum-sensing autoinducer 3-oxo-C12-homoserine-lactone, which is made by an enzyme regulated by LasR activity, inhibited phagocytosis and mitochondrial respiration, the phagocytosis resistance seen with *lasR* mutants appears to be bacterial cell intrinsic rather than due to secreted factors. Finally, we found that *lasR* LOF mutations altered the inflammatory profile upon infection of CF macrophages, with a shift from IL-1 family cytokine expression toward canonical inflammatory markers, including IL-6 and TNFα. Collectively, these data provide a potential explanation for both the prevalence of *lasR* mutants in the CF lung as well as their association with worse outcomes.

**IMPORTANCE:**

Cystic fibrosis (CF) is a genetically inherited disease that leads to chronic lung infections. *Pseudomonas aeruginosa* is often implicated in the worsening of lung disease, and it evolves in the lung over time to resist eradication. One of the most commonly disrupted genes in *P. aeruginosa* isolates from chronically infected CF lungs is *lasR*, which encodes a transcription factor that regulates multiple virulence factors. What contributes to the apparent fitness of *lasR* mutants in the CF lung is not well known. Our study shows that *lasR* loss-of-function mutants resist phagocytosis by macrophages, one of the fundamental mechanisms of clearance by the immune system. We identify mechanisms promoting resistance to phagocytosis and explore the downstream consequences on inflammatory responses. Understanding why *lasR* mutations arise could inform strategies to eradicate them from the CF lung and improve outcomes.

## INTRODUCTION

Cystic fibrosis (CF) is a multisystem disorder caused by mutations in the cystic fibrosis transmembrane conductance regulator (*CFTR*) gene, encoding a chloride and bicarbonate channel (1). In the lungs of people with CF (pwCF), CFTR mutations lead to dehydrated, sticky mucus and subsequent colonization with bacteria, often including *Pseudomonas aeruginosa* (2). Novel highly effective modulator compounds that improve trafficking and function of the CFTR protein have markedly improved outcomes for the subset of pwCF harboring CFTR mutations that are responsive to them (3–12); however, CFTR modulators cannot restore sterilizing immunity to the lungs, particularly when there is pre-existing structural damage (13, 14). Given their relatively recent availability, the long-term trajectory of lung function in pwCF remains unknown, even for those who are clinically improved on modulators.

Lung function over time is impacted by bacterial colonization, with cycles of infection and dysregulated inflammation leading to lung damage (1). The specific composition of the lung microbiota, as well as the host response to them, plays a role in determining whether lung function worsens or remains stable. Low bacterial diversity, *P. aeruginosa* colonization, and detection of *P. aeruginosa* with disruptions to the LasR/I quorum-sensing system have all been shown to correlate with accelerated lung function decline in pwCF (15–17).

LasR loss-of-function (LOF) mutants are one of the most commonly isolated morphotypes found in CF lungs (18). LasR LOF mutants have also been isolated from environments such as pond water and will arise *de novo* when *P. aeruginosa* are cultured *in vitro* (18–21). Their common emergence across various settings argues for a broad (or common) competitive advantage over LasR intact (LasR+) comparators; however, the reasons for selection *in vivo* are not entirely clear. Social cheating, whereby *lasR* LOF mutants can utilize secreted products from other *P. aeruginosa* isolates without incurring the metabolic cost of producing them, has been proposed as a mechanism for *lasR* mutant selection. The *lasR* mutation has also been shown to increase microoxic fitness and phosphate scavenging (22–24), providing additional possibilities for its evolutionary advantage. Indeed, *lasR* LOF mutants are selected for *in vitro* cultures due to a growth advantage in post-exponential phase (25–27); however, these experimental systems lack the host component. Interestingly, the phenotype of a lack of protease activity has been shown to increase in *P. aeruginosa* isolates that persist in pwCF on highly effective modulators, suggesting that *lasR* mutation may also be increased following modulator therapy (28).

In the CF lung, *P. aeruginosa* must coexist with host immune cells that are attempting to eradicate it, yet the effects of *lasR* LOF mutations on *P. aeruginosa* interactions with host immune cells remain comparatively unstudied. Studies on interaction with airway epithelial cells (29, 30), neutrophils (31, 32), and macrophages (33) have revealed variable effects on inflammatory responses. Macrophages are responsible for orchestrating the host immune response in the lung, phagocytosis of pathogens and debris, and have been shown to have altered inflammatory and effector activity in CF (34). Although CFTR modulators have been shown to improve CF macrophage function (35–37), an improved mechanistic understanding of how modulators affect the host immune response could benefit the majority of pwCF who are now eligible for highly effective modulator treatment and lead to novel interventions that might reduce bacterial persistence.

We hypothesized that loss of LasR function would alter interactions with host macrophages as a mechanism of *lasR* mutant persistence within the CF lung, and that CFTR modulators would further impact host response. We found that *lasR* LOF mutants indeed resist phagocytosis in multiple *P. aeruginosa* backgrounds, including laboratory and clinical isolates. Phagocytosis resistance was maintained in mixed cultures with LasR+ relatives. The *lasR* mutant’s resistance to phagocytosis by primary CF monocyte-derived macrophages (MDMs) was maintained despite the presence of highly effective CFTR modulators. Finally, the *lasR* mutation shifted the inflammatory response of MDMs to *P. aeruginosa* infection away from IL-1 family cytokines toward canonical proinflammatory cytokines IL-6 and TNFα. Collectively, these results highlight the importance of understanding *P. aeruginosa lasR* LOF in the context of immune cells and provide potential explanations for its increased prevalence and virulence in CF. Some of these results have previously been presented in the form of abstracts (38–42).

## RESULTS

### *lasR* loss-of-function mutations confer phagocytosis resistance independent of the background *P. aeruginosa* strain

**Fig 1:**
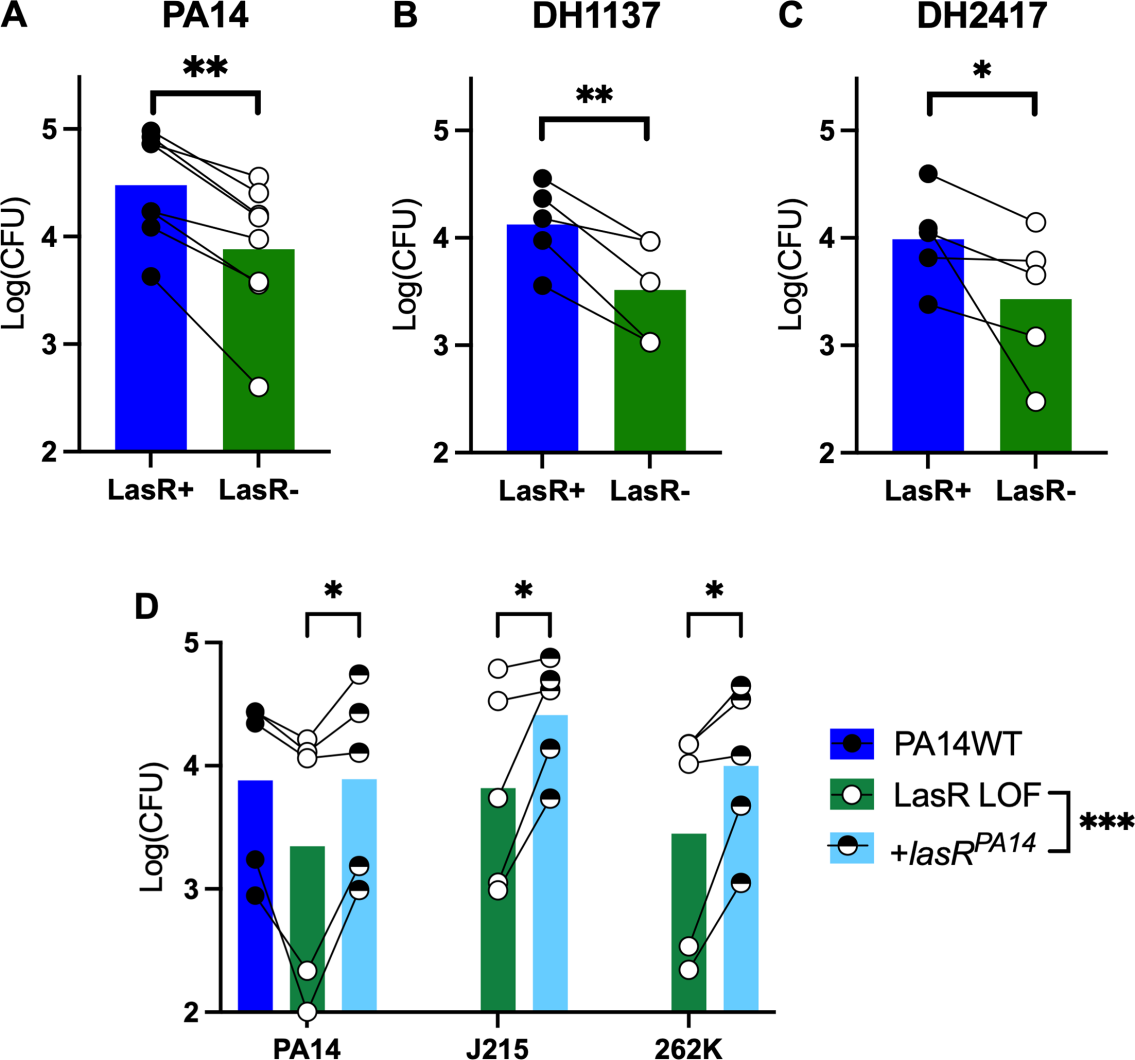
*lasR* mutation confers phagocytosis resistance independent of parental strain. (**A**) THP-1 cells were differentiated into macrophages by the addition of 50 nM phorbol 12-myristate 13-acetate for 48 hours. Phagocytosis was then measured by a gentamicin protection assay at 20 minutes of bacterial uptake. After the removal of extracellular bacteria by washing and adding gentamicin, intracellular colony-forming units were quantified by plating onto LB agar. Phagocytosis of PA14 wild type and PA14 ∆*lasR* (LasR−) was assessed in eight independent assays. Log-transformed means of three technical replicates per assay are displayed. **P < 0.01 for PA14 WT vs LasR− by mixed model linear regression. (**B**) Identical assays to panel A were run using naturally occurring paired LasR+/− clinical isolates of *P. aeruginosa* derived from the same parental strain DH1137. **P < 0.01 for PA14 WT vs LasR− by mixed model linear regression. (**C**) Identical assays to panel A were run using naturally occurring paired LasR+/− clinical isolates of *P. aeruginosa* derived from the same parental strain DH2417. **P < 0.01 for PA14WT vs LasR− by mixed model linear regression. (**D**) Similar assays to panels A–C were run using *P. aeruginosa* LOF mutant strains that were then complemented with LasR. Means of two to four technical replicates from five independent assays are shown. *P < 0.05 for each LasR complementation individually, with ***P < 0.001 for LasR complementation globally by mixed model linear regression.

To test the hypothesis that loss-of-function mutations in *lasR* confer phagocytosis resistance, we utilized model THP-1 monocytes, which can be differentiated into macrophage-like cells by the addition of the protein kinase C activator phorbol 12-myristate 13-acetate (43). We performed gentamicin protection assays with pairs of LasR+ and LasR− *P. aeruginosa* to compare rates of bacterial uptake. In these assays, macrophages are washed to remove antibiotics from the medium, then allowed to phagocytose bacteria. After the phagocytosis period, extracellular bacteria are washed away, and high-concentration gentamicin is re-added to the milieu. Gentamicin does not penetrate the eukaryotic membrane; therefore, phagocytosed bacteria are “protected.” At that point, cells are washed again, and then macrophages are lysed for the quantification of phagocytosed bacteria. We chose a 20-minute time point to study phagocytosis as this provides an investigation of uptake alone, whereas longer time points may see a mixture of phagocytosis and killing (36). In laboratory strain PA14, the ∆*lasR* mutant in THP-1 co-culture had fewer cells that were phagocytosed than in the otherwise isogenic wild-type (WT) strain (Fig. 1A). We then utilized two closely related pairs of LasR+/LasR− clinical isolates, in which *lasR* LOF evolved over the course of infection to confirm this effect with *P. aeruginosa* derived from the CF lung. LasR LOF continued to confer phagocytosis resistance in both clinical isolate backgrounds (Fig. 1B and C), demonstrating that this was not a strain-specific effect. *P. aeruginosa* motility has been shown to enhance phagocytosis (44); therefore, we tested whether LasR LOF impacted motility. We found no differences between PA14 WT and paired LasR+/LasR− clinical strains with regard to swimming motility as a measure of flagellar function (Fig. S1A) or twitching motility, which indicates the function of type IV pili (Fig. S1B), suggesting that the differences in phagocytosis efficiency seen in Fig. 1 were not related to motility. While genome analyses of DH2417 and DH2415 revealed multiple differences (see Dataset S1), no differences in genes known to impact phagocytosis of *P. aeruginosa* were found. As attempts to complement DH2415 with the *lasR* from strain PA14 (which has functional LasR) were not successful, genetic complementation experiments were performed with strain PA14 and two other previously characterized LasR− clinical isolates (24, 45). We found consistently that the addition of the *lasR^PA14^* allele to mutant strains increased phagocytosis (Fig. 1D), further supporting the hypothesis that LasR LOF itself is responsible for altering phagocytosis susceptibility. Finally, there can be differences between opsonic vs non-opsonic phagocytosis (46); therefore, we performed additional assays with *P. aeruginosa,* which were pre-incubated with human serum (47, 48) (Fig. S2). We found that *lasR* mutants continued to display lower phagocytosis levels when compared with *lasR-*WT strains.

### LasR, but not RhlR, autoinducer homoserine lactone inhibits phagocytosis

**Fig 2:**
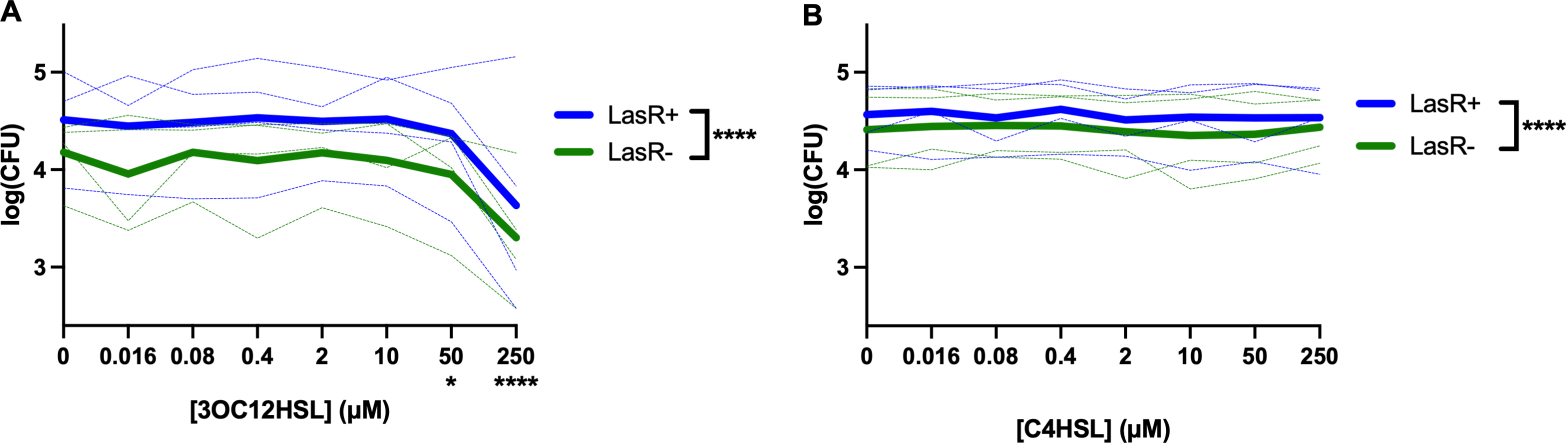
LasR-regulated autoinducer inhibits phagocytosis in a dose-dependent manner. (**A**) THP-1 macrophages were incubated with the indicated concentration of 3OC12HSL for 20 minutes prior to performing a gentamicin phagocytosis assay with PA14 WT and PA14∆*lasR* (LasR−) in parallel. Four separate experiments were performed with three technical replicates each. The means of technical replicates from each individual experiment are shown in the light lines, with darker lines at the means of the four experiments. *P < 0.05 and ****P < 0.0001 for 50 and 250 μM 3OC12HSL relative to DMSO, respectively, and LasR+ vs LasR− by mixed model linear regression. (**B**) Identical assays to panel A were performed with C4HSL in place of 3OC12HSL. ****P < 0.0001 by mixed model linear regression.

One of the primary signaling molecules regulated by LasR is the autoinducer N-(3-oxododecanoyl)-L-homoserine lactone (3OC12HSL) that binds to the LasR regulator to induce a positive feedback cycle. 3OC12HSL has been shown to have direct effects on eukaryotic metabolism and signaling (49–60), as well as enhancing phagocytosis of yeast (61). The local concentrations of HSLs *in vivo* are not known. While nanomolar concentrations have been detected in CF sputum (62), HSLs are rapidly degraded by paraoxonases produced by mammalian epithelial cells (63, 64); therefore, sputum concentrations may underestimate local HSL concentrations in the lung. Other investigators report activity of HSLs in the 50−100 μM range (50, 55, 58, 59), and local concentrations as high as 600 μM have been found in *P. aeruginosa* biofilms (65). We therefore tested whether 3OC12HSL, over a broad range of concentrations from 0.4 to 250 μM, would increase the phagocytosis of *P. aeruginosa* as an explanation for the decreased *lasR* mutant phagocytosis. Contrary to this hypothesis, we found that 3OC12HSL inhibited phagocytosis of both LasR+ and LasR− PA14 at concentrations of 50 μM or higher in a dose-dependent manner (Fig. 2A). By comparison, the RhlR autoinducer C4-homoserine lactone (C4HSL) had no appreciable effect (Fig. 2B). Under all concentrations of HSLs tested, there continued to be a reproducible difference between LasR+ and LasR− phagocytosis. These results suggest that 3OC12HSL is not responsible for the effects seen in Fig. 1.

### Phagocytosis resistance of LasR− strains is maintained in mixed cultures

**Fig 3:**
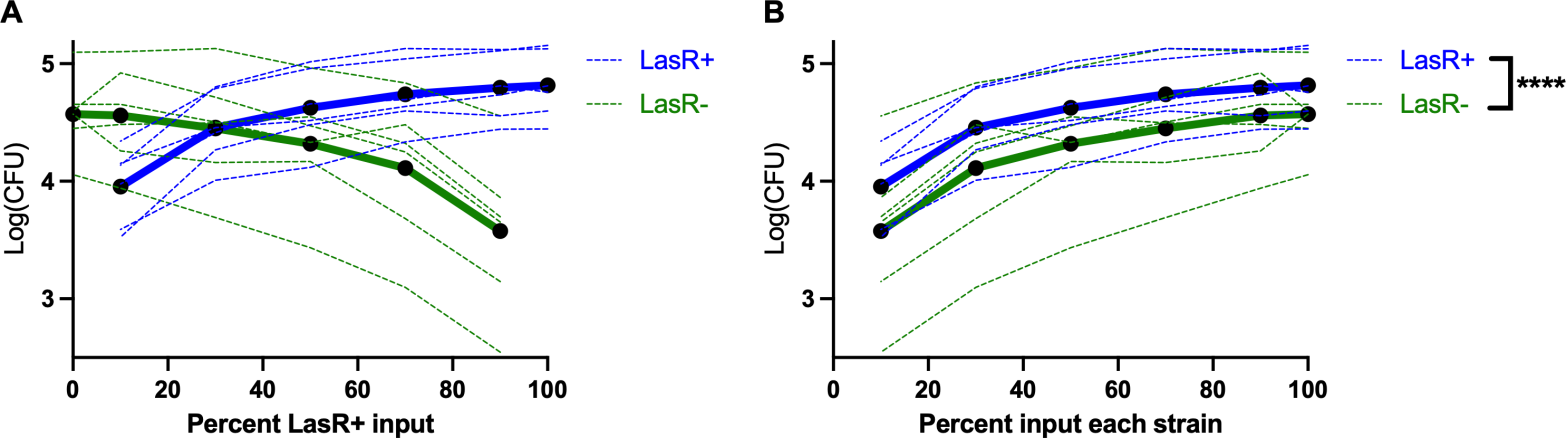
*lasR* mutation confers phagocytosis resistance in mixed cultures. (**A**) A PA14∆*lasR* strain engineered to express a LacZ reporter was mixed with PA14 wild type at 0%, 10%, 30%, 50%, 70%, 90%, and 100% with a fixed total multiplicity of infection of 10 for the two strains, and a phagocytosis assay was performed as previously. After lysis, bacteria were plated onto X-gal plates to allow for differentiation of colony genotype. Means of three technical replicates from six independent experiments are graphed, with a dark line at the mean of the six experiments. (**B**) The same data as in panel A are graphed relative to the percentage of each strain input, rather than percent LasR− to better visualize differences. ****P < 0.0001 by mixed model linear regression.

We, therefore, sought to ascertain whether *lasR* mutation effects were due to secreted or cell-intrinsic factors. We performed phagocytosis assays with a mixture of LasR+/LasR− PA14 derivatives at varying percentages (0/100, 10/90, 30/70, 50/50, 70/30, 90/10, and 100/0). If secreted factors were responsible for the phagocytosis resistance, we would, for example, expect that in a 50:50 mixture of LasR+/LasR− bacteria, there would be no difference in rates of phagocytosis, with proportional effects at different ratios. The LasR− competitor in the PA14 background bore a *lacZ* reporter gene, and plating onto X-gal plates allowed for post-assay determination of strain identities when mixed with an untagged wild type. While we found that bacterial uptake was generally proportional to the percentage of input for each strain (Fig. 3A), there was a persistently lower amount of LasR− *P. aeruginosa* phagocytosed. Figure 3B displays the same data as in Fig. 3A graphed relative to the percentage of input for each strain such that the decreased phagocytosis of LasR− strains is evident. These data suggest that the effects of *lasR* mutation on phagocytosis are due to cell intrinsic rather than secreted factors.

### Primary human macrophages augment phagocytosis with CFTR modulators, but LasR phagocytosis resistance is maintained

**Fig 4:**
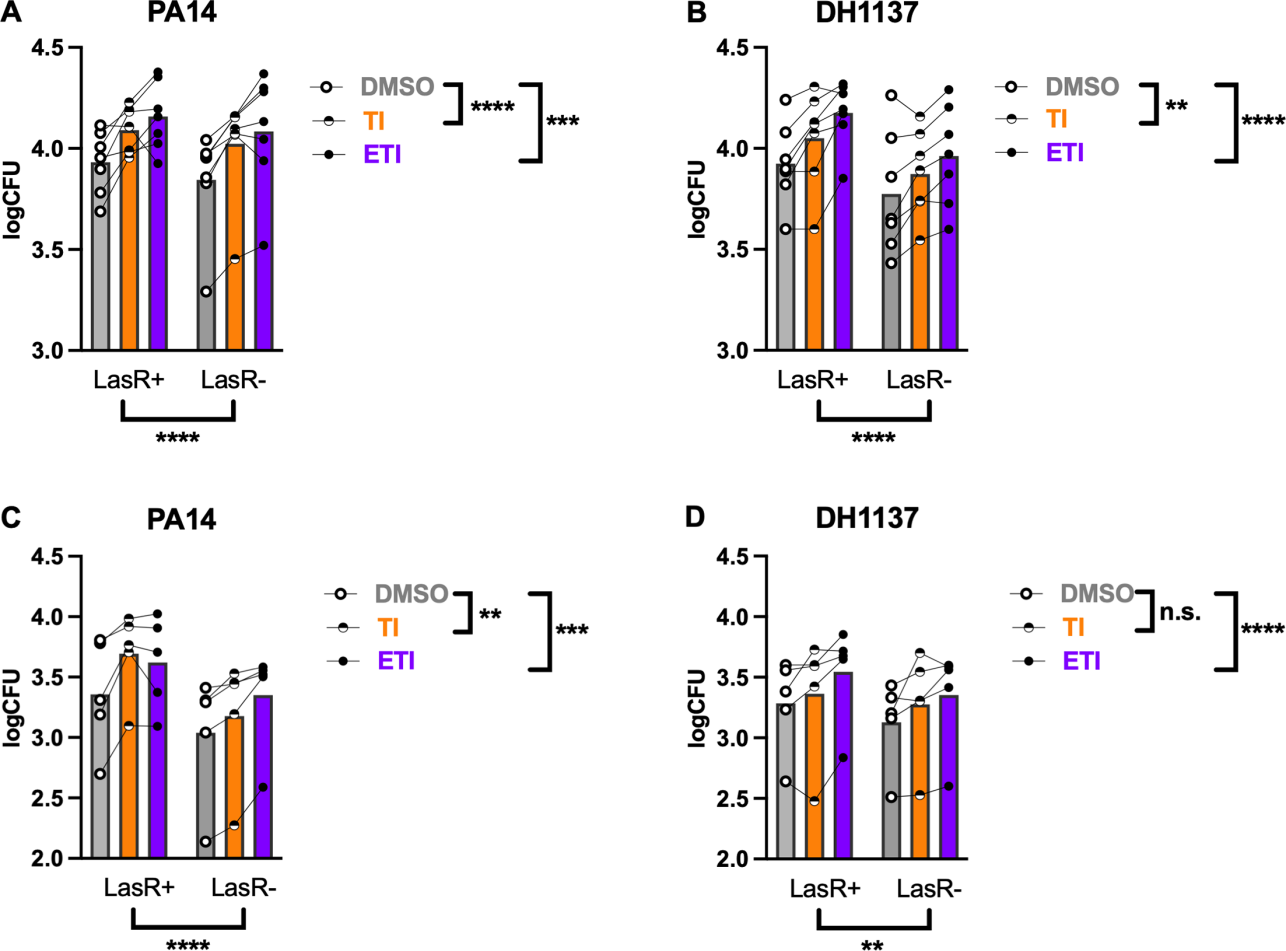
*lasR* mutants exhibit reduced phagocytosis by primary MDMs irrespective of CFTR genotype or modulators. (**A**) CF MDMs were treated for 48 hours with CFTR modulators as indicated, then infected with PA14 WT or PA14 *∆lasR* (LasR−)*.* Lines connecting dots represent data from a single subject (*n* = 7). (**B**) As in panel A, except the clinical isolate DH1137 and its derivative *lasR* mutant were used. (**C**) Non-CF MDMs (*n* = 5) were infected with PA14 WT or LasR- derivative as in panel A. (**D**) Non-CF MDMs were infected with DH1137 or its *lasR* mutant derivative as in panel B. **P < 0.01, ***P < 0.001, and ****P < 0.0001 for indicated comparisons (global effects of each variable within a given assay) by mixed model linear regression. Data were combined with previously published data on LasR+ strains only (37).

To determine if the differences between phagocytosis of LasR+ and LasR− cells seen in THP-1 cells were also observed when using primary human cells, we isolated peripheral blood-derived mononuclear cells from volunteers and differentiated them into monocyte-derived macrophages *in vitro* for 7 days. We first tested cells from CF volunteers. We and others have demonstrated that CFTR modulators have a positive effect on macrophage and monocyte phagocytosis (36, 37, 66–70), and as a majority of pwCF are now eligible for CFTR modulators, it is important to understand whether they may play an additional role in modifying the response to *lasR* mutants. We tested one pair of laboratory isolates and one pair of clinical isolates (DH1137/6) in MDMs that were treated for 48 hours with CFTR modulators or DMSO as a control. The PA14∆*lasR* (LasR−) strain was less efficiently phagocytosed by primary CF MDMs relative to its LasR+ progenitor (Fig. 4A). Tezacaftor/ivacaftor (TI) and elexacaftor/tezacaftor/ivacaftor (ETI) improved phagocytosis of both strains in the PA14 background (Fig. 4A); however, there was no statistically significant interaction with *lasR* status. We found similar results using paired clinical *P. aeruginosa* isolates, where *lasR* mutants were less efficiently phagocytosed, and CFTR modulators improved phagocytosis of both strains with no difference in response to modulators between isolates (Fig. 4B). We have previously demonstrated that non-CF MDMs respond similarly to CFTR modulators when compared with CF MDMs (37); therefore, we tested whether any of the effects on *lasR* mutant phagocytosis might be modified by *CFTR* genotype. Non-CF MDMs displayed a similar pattern, with decreased phagocytosis of *lasR* mutants, improved phagocytosis in the presence of CFTR modulators, and no statistically significant interaction between modulators and *lasR* mutation (Fig. 4C). With the LasR+/LasR− clinical isolates, there continued to be an effect of *lasR* mutation (Fig. 4D); however, double CFTR modulator treatment only displayed a non-significant trend toward enhanced phagocytosis, with triple modulators still proving effective (Fig. 4D). Collectively, these results confirm that *lasR* mutants exhibit reduced phagocytosis by primary human CF and non-CF macrophages, and that while CFTR modulator treatment is still beneficial, it fails to restore phagocytosis to the level of LasR+ strains.

### *lasR* mutation abrogates the inhibitory effect of *P. aeruginosa* conditioned medium on macrophage mitochondrial respiration

**Fig 5:**
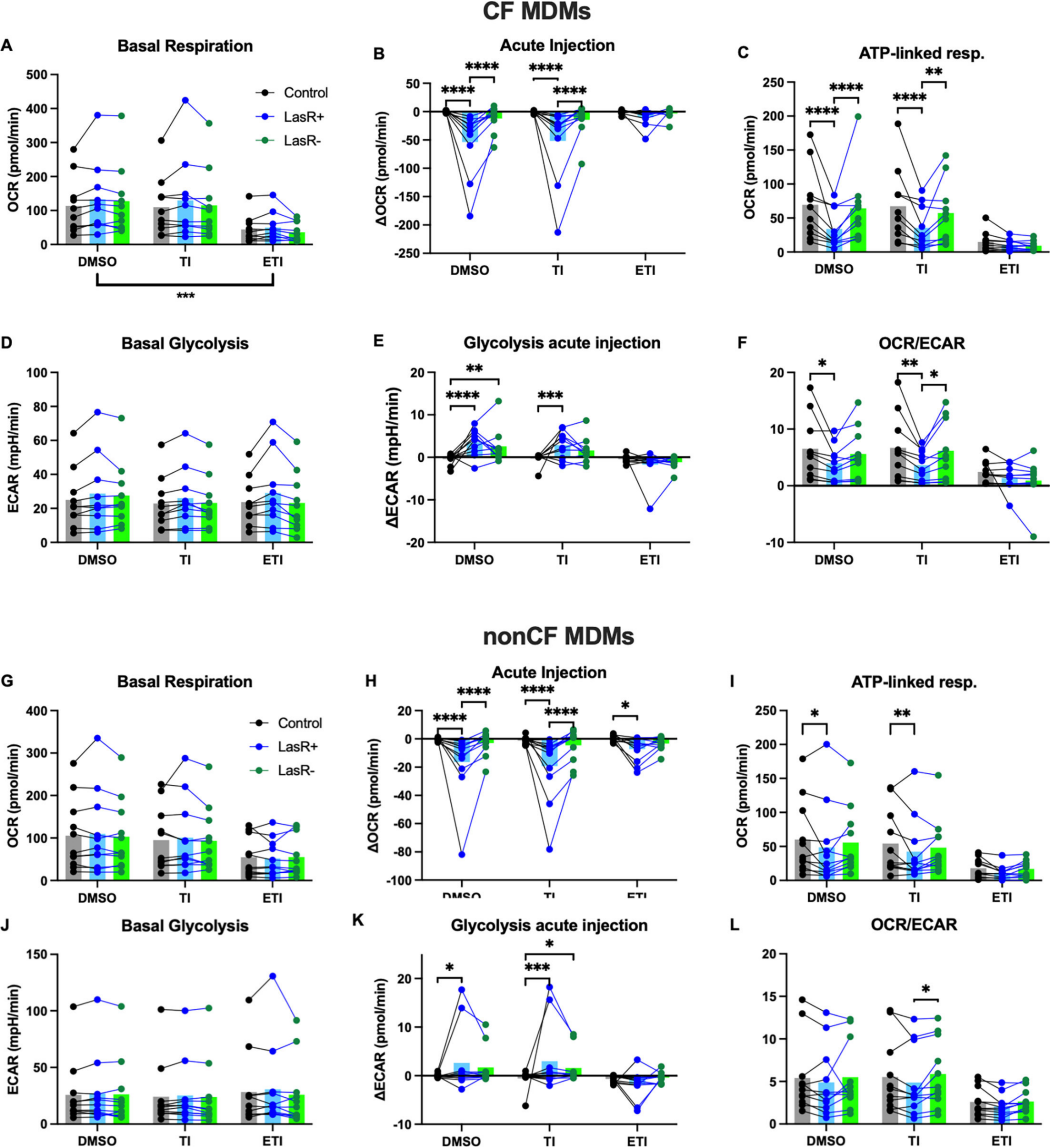
Effects of CFTR modulators and LasR+/− secreted products on macrophage metabolism. CF and non-CF MDMs were differentiated with M-CSF and then treated for 48 hours with CFTR modulators. A Seahorse mitostress assay with acute injection of *P. aeruginosa* conditioned medium was then performed. (**A–F**) Calculated parameters as indicated based on the mitostress assay of CF MDMs (*n* = 10). Each point with connecting lines represents the mean of technical replicates from an individual subject. Bars represent the means of all the subjects within the group. (**G–L**) Calculated parameters as indicated for non-CF MDMs (*n* = 11). *P < 0.05, **P < 0.01, ***P < 0.001, and ****P < 0.0001 for indicated comparisons by mixed model linear regression. Data were combined with previously published data on LasR+ strains only (37).

Macrophage metabolism impacts their inflammatory predilection, with activated macrophages relying on glycolysis for ATP generation, whereas pro-resolution utilized aerobic respiration (71, 72). We have previously demonstrated that secreted products from *P. aeruginosa* inhibit aerobic respiration in MDMs (37), and we hypothesized that the *lasR* mutation may impact this effect. In addition, we saw that ETI inhibits aerobic respiration without significantly affecting glycolysis. We therefore performed Seahorse metabolic flux analysis on CF MDMs pre-treated with CFTR modulators, and then conditioned media from either LasR+ or LasR− PA14 strains were added acutely. Oxygen consumption rate (OCR) was measured as a proxy for mitochondrial respiration, and extracellular acidification rate (ECAR) as a proxy for lactate generation/glycolysis. Figure S3A through C demonstrates example readouts of OCR from a single CF subject pre-treated with DMSO, TI, or ETI, respectively, and Fig. S3D through F are the corresponding readouts for ECAR. Addition of oligomycin, FCCP, and rotenone/antimycin A allows for the measurement of additional parameters related to mitochondrial respiration. In CF MDMs, ETI decreased basal respiration similarly to prior experiments, whereas TI had no effect (Fig. 5A). Upon acute injection of LasR+ conditioned medium, there was a decrease in mitochondrial respiration (Fig. 5B); however, LasR− conditioned medium completely lost this effect. In the setting of low basal mitochondrial respiration with ETI, the effect of *P. aeruginosa* conditioned medium (either LasR+ or LasR−) was lost (Fig. 5B). Corresponding decreases in ATP-linked respiration were seen with LasR+ conditioned medium and ETI pre-treatment (Fig. 5C). Basal glycolysis was not affected by CFTR modulators or different prior to injection (Fig. 5D); however, LasR+ but not LasR− conditioned medium caused an acute rise in glycolysis (Fig. 5E). This effect was lost upon ETI pre-treatment (Fig. 5E). OCR/ECAR ratio is a way of gauging relative contributions of each pathway to ATP generation, and in line with the effects seen on ATP-linked respiration and glycolysis, OCR/ECAR was decreased with LasR+ but not LasR− conditioned media, as well as by ETI (Fig. 5F). Measurements of maximal respiration, spare respiratory capacity, non-mitochondrial respiration, and proton leak are shown in Fig. S4. These results collectively demonstrate that the *lasR* mutation eliminates the mitochondrial effects of *P. aeruginosa* exoproducts. Additionally, CFTR modulators inhibit basal mitochondrial activity, and there is no additive effect of *P. aeruginosa* exoproducts. Both of these observations have potential downstream implications for the induction of inflammation.

We next examined non-CF MDMs to determine whether there may be any differences in how they respond to *lasR* mutants. Similarly to CF MDMs, we found the ETI inhibits basal respiration (Fig. 5G) with no effect of TI, in agreement with our prior published work (37). LasR+ but not LasR− conditioned medium inhibited mitochondrial respiration (Fig. 5H) and decreased ATP-linked respiration (Fig. 5I). These effects were decreased or eliminated by ETI pretreatment. Modulators had no effect on basal glycolysis (Fig. 5J), with increased glycolysis secondary to LasR+ but not LasR− conditioned medium (Fig. 5K). In contrast to CF MDMs, differences in OCR/ECAR were not statistically significant (Fig. 5L). Measurements of maximal respiration, spare respiratory capacity, non-mitochondrial respiration, and proton leak are shown in Fig. S5.

### LasR autoinducer homoserine lactone inhibits macrophage mitochondrial respiration and induces glycolysis

**Fig 6:**
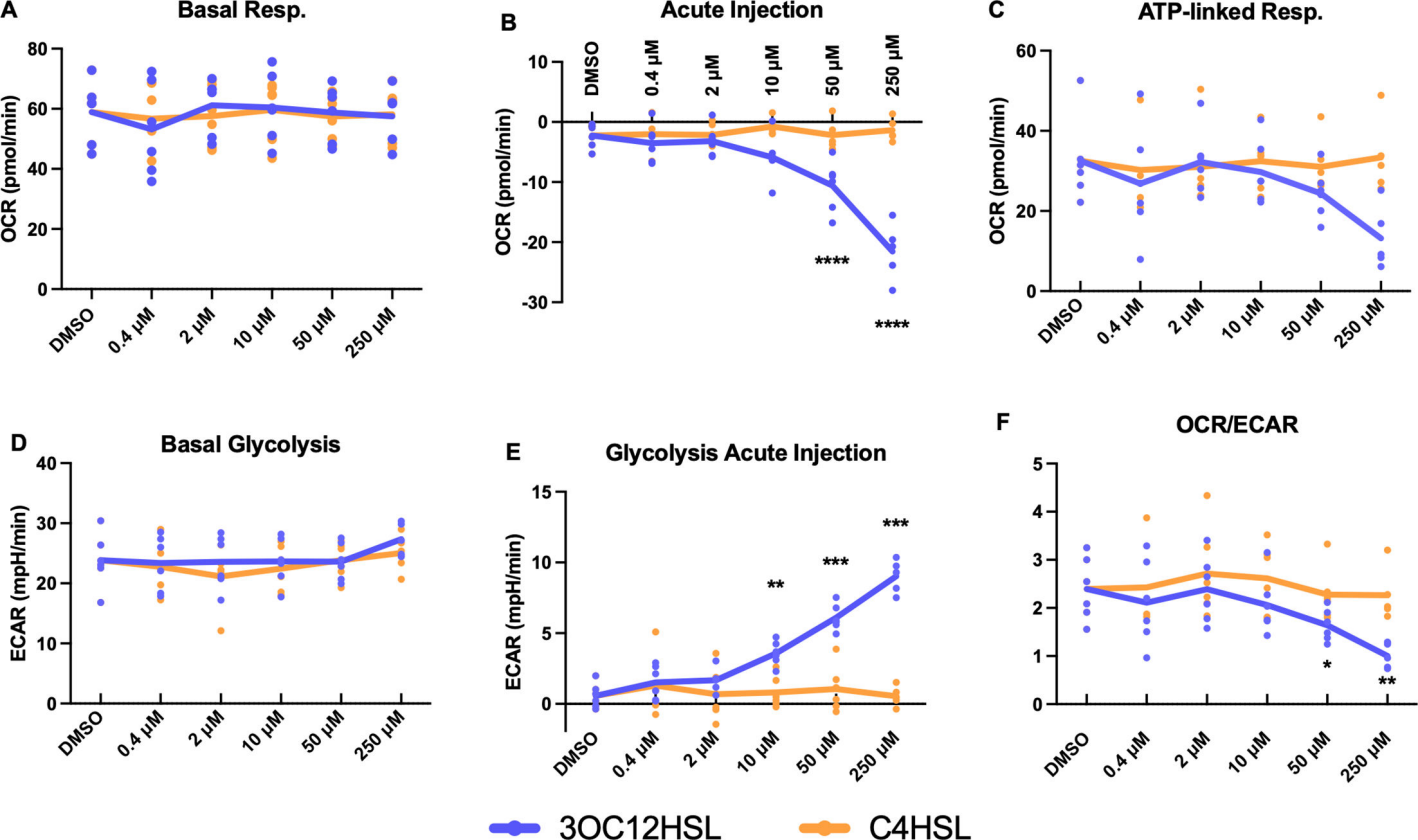
3OC12HSL inhibits mitochondrial respiration in THP-1 macrophages. (**A–F**) A Seahorse mitostress assay was run with acute injection of various concentrations of 3OC12HSL or C4HSL. Parameters were calculated as per standard protocol. Points represent averages of six to eight technical replicates from six individual experiments, with solid lines at the global means. *P < 0.05, **P < 0.01, ***P < 0.001, and ****P < 0.0001 for the indicated comparisons relative to DMSO-treated wells by mixed model linear regression.

The divergence between LasR+ and LasR− conditioned medium effects on MDM metabolism led us to hypothesize that 3OC12HSL might underlie these effects. To test this, we returned to THP-1-derived model macrophages and performed Seahorse metabolic analysis with acute injection of 3OC12HSL, with C4HSL as a control. After first confirming no differences in basal respiration prior to injection (Fig. 6A), we found a dose-responsive effect of 3OC12HSL injection (Fig. 6B), causing inhibition of mitochondrial respiration, whereas C4-HSL had no effect. This was partially reflected in differences in ATP-linked respiration (Fig. 6C), although the effects were not statistically significant. Simultaneous measurement of glycolytic rates confirmed no basal differences in glycolysis (Fig. 6D), with a dose-dependent increase in glycolysis upon 3OC12HSL injection (Fig. 6E) and no effect of C4HSL. The shift in metabolic profile from mitochondrial respiration to glycolysis by 3OC12HSL was confirmed with decreased OCR/ECAR (Fig. 6F). Measurements of maximal respiration, spare respiratory capacity, non-mitochondrial respiration, and proton leak are shown in Fig. S6. HSLs displayed no measurable cytotoxicity to THP-1 macrophages as measured by LDH release (Fig. S7). These results suggest that 3OC12HSL may underlie the effects of LasR+ *P. aeruginosa* exoproducts on mitochondrial respiration.

### *lasR* mutation shifts the inflammatory cytokine profiles of primary human macrophages away from IL-1 family cytokines toward IL-6 and TNFα, irrespective of CFTR modulators and *CFTR* mutation status

**Fig 7:**
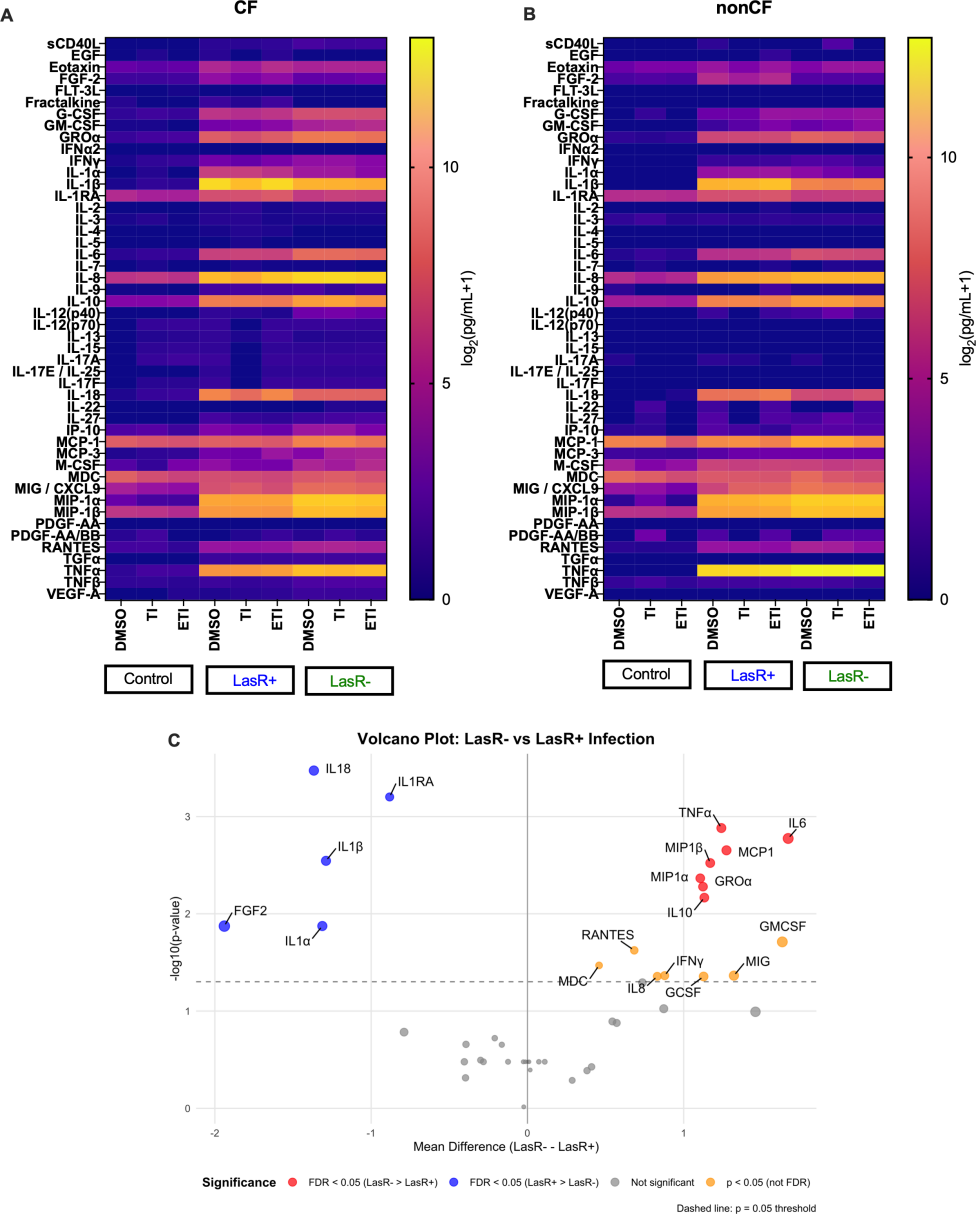
*lasR* mutation alters inflammatory cytokine expression profile by CF and non-CF MDMs. (**A**) CF MDMs (*n* = 11 subjects) were pre-treated with CFTR modulators and then infected with DH1137 (LasR+) or DH1136 (LasR−) for 2 hours, then cells were washed, and media were replaced. Supernatants were collected 2 hours after washing and analyzed by cytokine multiplex assay. Means of log-transformed data are graphed. (**B**) Non-CF MDMs (*n* = 4) were infected in the same way, and cytokines were analyzed by multiplex. (**C**) Volcano plot of fold difference between LasR+ and LasR− cytokine results, DMSO-treated group only. Red dots represent cytokines that were higher in LasR− infection, and blue dots represent those that were higher in LasR+ infection, after adjustment for multiple comparisons (false discovery rate < 0.05). Yellow dots represent cytokines with *P* < 0.05; however, they were no longer statistically significant after adjustment for multiple comparisons.

Finally, we investigated whether the *lasR* mutation impacted the inflammatory profiles of MDMs and the interplay with CFTR modulators. We pretreated MDMs with CFTR modulators for 48 hours, then infected MDMs for 2 hours with LasR+ or LasR− clinical isolates of *P. aeruginosa* and performed cytokine multiplex analysis on the supernatants. Unsurprisingly, we found broad induction of multiple inflammatory cytokines upon *P. aeruginosa* infection of CF MDMs (Fig. 7A). There was little appreciable effect of CFTR modulators, consistent with our prior work and that of others (37, 73). Non-CF MDMs had a similar inflammatory response to CF MDMs, with no statistically significant differences between the groups (Fig. 7B). We then combined results and tested specifically for differences between LasR+ and LasR− infection. IL-1 family cytokines IL-1α, IL-1β, and IL-18 were higher with LasR+ infection, whereas IL-6, IL-10, MCP-1, MIP-1α, and MIP-1β were higher with LasR− infection (Fig. 7C), demonstrating a specific alteration in the inflammatory profile after macrophage response to *lasR* mutants. *P. aeruginosa* can be cytotoxic to mammalian cells via delivery of effectors, including those regulated by LasR (74, 75); therefore, we tested whether there was evidence of cytotoxicity in our assays. We confirmed that these differences were not explained by variable cytotoxicity by measuring lactate dehydrogenase (LDH) release. We found that LDH levels were generally low (at or below the amount measured in media control), with no differences between infected and uninfected samples, or between LasR+ and LasR− clinical strains (Fig. S8).

## DISCUSSION

Loss-of-function mutations in *lasR* have been demonstrated to be important for *P. aeruginosa* persistence and virulence within the CF lung (15, 18). How this impacts interactions with host immune cells, specifically in the presence of highly effective CFTR modulators, is incompletely understood (29–33, 76). We have identified for the first time a robust and reproducible resistance to phagocytosis by macrophages that is hypothesized to contribute to persistence in the CF lung. While the difference was modest (0.3–0.5 log depending on the assay), this represents a relative difference of two- to threefold uptake in 20 minutes; therefore, over the years, this could represent a significant evolutionary pressure. The phagocytosis resistance was present in multiple background strains, including a common laboratory strain (PA14) and two pairs of clinical isolates from the lungs of pwCF (Fig. 1), increasing the generalizability of our findings. Complementation assays further implicated LasR itself as the causative factor underlying phagocytosis susceptibility (Fig. 1D).

We also identified potential factors by which LasR mutants resist phagocytosis. The LasR autoinducer 3OC12HSL inhibited *P. aeruginosa* phagocytosis (Fig. 2), in contrast to prior studies of yeast phagocytosis (61). If 3OC12HSL were responsible for differences in phagocytosis, however, it would be expected that LasR mutants would exhibit *higher* phagocytosis rates, which was the opposite of what was seen. Differences in phagocytosis rates were also maintained between LasR+ and LasR− strains when they were mixed in different ratios (Fig. 3). While this does not rule out locally secreted factors (directly at the interface between phagocyte and bacterium), it suggests that broadly secreted factors activating or inhibiting macrophages are not responsible, as they would be expected to impact phagocytosis of each strain equally.

We also explored the interplay between CFTR modulators and *lasR* mutation with phagocytosis and inflammation in primary MDMs. While CFTR modulators were able to enhance phagocytosis of all strains tested, consistent with our prior work (37), the decrement with *lasR* mutation was maintained (Fig. 4). Given the known persistence of *P. aeruginosa* in the lungs of pwCF despite highly effective modulator therapy (14), this is relevant to understanding *P. aeruginosa* colonization. We previously observed that *P. aeruginosa* secreted products inhibited mitochondrial respiration in MDMs (37), as did the triple modulator combination ETI. Intriguingly, we found that the *lasR* mutation essentially eliminated this effect (Fig. 5). In combination with the effects of exogenous 3OC12HSL (Fig. 6), this suggests that the acyl-homoserine lactone is the responsible factor, in line with previous studies of epithelial cells and fibroblasts (50, 51, 53–56, 77).

Unsurprisingly, given the variable rates of phagocytosis and differences in effects on mitochondrial function in MDMs, we found that *lasR* mutation impacted inflammatory cytokine profiles upon infection of CF and non-CF MDMs (Fig. 7). While infection generally induced a broad inflammatory cytokine profile, there was a shift from IL-1 family cytokines IL-1α, IL-1β, IL-1RA, and IL-18 toward more classical inflammatory markers IL-6 and TNFα, as well as macrophage inflammatory protein 1α and 1β, and macrophage chemoattractant protein 1. There was a trend toward increased IL-8 for *lasR* mutant infection that was not statistically significant after adjustment for multiple comparisons (Fig. 7C). A shift toward IL-1 family cytokines may reflect increased inflammasome activation versus direct proteolytic activation of IL-1 family precursor proteins. The LasR-regulated elastase LasB has previously been demonstrated to directly activate pro-IL-1β in mice and human macrophages (78, 79), consistent with our data showing less mature IL-1β for infections with *lasR* loss-of-function mutants (Fig. 7C). Early CF lung *P. aeruginosa* isolates have been shown to be more potent inflammasome activators when compared to later isolates; however, LasR functional status was not tested in the study by Phuong et al. (80). Decreased protease expression has been seen in persistent *P. aeruginosa* isolates in those on ETI, also suggesting *lasR* mutation may be increased post-modulators (28). It is important to note that in our system, significant LasR quorum-sensing activation would not necessarily be expected, as bacteria are sub-cultured at low density prior to addition to cells, and cells were infected for 2 hours. In contrast to its effect of increasing processed IL-1β, LasB can decrease IL-8 levels upon infection of airway epithelial cells via direct digestion of IL-8 (81). Future experiments will help elucidate the exact mechanism by which *lasR* mutation alters the inflammatory cytokine milieu in the shorter term. We are also currently conducting transcriptomic studies on macrophages infected in parallel with LasR+ or LasR− isolates, which should shed light on whether the effects are transcriptional versus post-transcriptional.

Strengths of our work include analysis of *lasR* mutations in multiple backgrounds, including a common laboratory strain, as well as naturally occurring clinical isolates, to improve the generalizability. We began to investigate the mechanisms by which *lasR* loss-of-function mutants may confer phagocytosis resistance, although work remains to further elucidate the full mechanism. We confirmed the results found in a human cell line (THP-1 cells) with primary human macrophages from multiple subjects and investigated the interplay with CFTR modulators and genotype (mutant vs wild-type CFTR). Studies performed in parallel on cells from the same subject allowed for a close interrogation of the effects of CFTR modulators and LasR mutation on phagocytosis, metabolism, and inflammation.

Our work also carries several limitations. MDMs, while they are a commonly used surrogate for primary lung macrophages due to their ease of procurement, do not behave identically to lung macrophages (82). We plan to expand our work with primary lung macrophages isolated via bronchoscopy (83) and compare them with classically generated MDMs as well as the novel differentiation protocol proposed by Pahari et al. (82). Additionally, while our *ex vivo* studies provided the opportunity to directly compare the effects of LasR status and modulators in paired samples from the same subject, they are necessarily reductive and cannot replicate *in vivo* conditions. Mouse models differ from human CF in chronicity and acuity of lung infection; however, there are data suggesting that *lasR* mutants are more virulent in mice as well (31, 81). Finally, we found no direct effect of CFTR modulators, including ETI, on inflammatory responses by MDMs, in contrast to human studies pre- and post-modulator, which demonstrate decreased inflammation (14, 68, 84–87). *In vivo* studies, however, cannot separate direct effects on immune cells from decreased mucus and pathogen burden, leading to a systemic decrease in inflammation.

In summary, the *lasR* mutation confers reproducible resistance to phagocytosis, altered metabolic effects on macrophages, and a change in inflammatory cytokine repertoire upon infection. These effects were independent of the background strain of *P. aeruginosa*, as well as the CFTR genotype and CFTR modulator therapy. Our work adds to the body of literature on how and why *lasR* mutants are problematic in the lungs of pwCF and will likely continue to be so in the highly effective modulator era.

## MATERIALS AND METHODS

### Source of key materials and bacterial strains

Commercial sources or references are provided in Table 1.

**TABLE 1:** Source of key chemicals and bacterial strains

Chemical or strain	Source of materials	Catalog number
M-CSF	Miltenyi	130-096-491
Pan monocyte isolation kit	Miltenyi	130-096-537
3-Oxo-C12-homoserinelactone	Sigma	O9139
C4-homoserinelactone	Chemodex	B0267
Ivacaftor	Selleckchem	S1144
Tezacaftor	Selleckchem	S7059
Elexacaftor	Selleckchem	S8851
Seahorse mitostress kit	Agilent	
THP-1 cells	ATCC	TIB-202
Phorbol 12-myristate 13-acetate	Sigma	P1585
PA14 WT	(88)	Hogan Lab (DH122)
PA14 ∆*lasR*	(89)	Hogan Lab (DH164)
PA14 ∆*lasR* att::P*rhlI-lacZ*	(90)	Hogan Lab
DH1137 (LasR+)	(91)	Hogan Lab (DH1137)
DH1136 (LasR−)	(91)	Hogan Lab (DH1136)
DH2417 (LasR+), NC-AMT0101-1-2 (parent of NC-AMT0101-1)	(91)	Hogan Lab (DH2417)
DH2415 (LasR−), NC-AMT0101-1-1, related to DH2417 with LasR LOF (frameshift) allele	(91)	Hogan Lab (DH2415)
DH4740 PA14LasR− +*lasRPA^PA14^*	(45)	Hogan Lab (DH4740)
DH2403 J215 (LasR−)	(45)	Hogan Lab (DH2403)
DH2484 J215 +lasRPA^PA14^	(45)	Hogan Lab (DH2484)
DH2590 262K (LasR−)	(24)	Hogan Lab (DH2590)
DH2743 262K + lasRPA^PA14^	(24)	Hogan Lab (DH2743)

### THP-1 cell culture

THP-1 cells were cultured in RPMI with 10% FBS, 50 µM β-mercaptoethanol, and 50 μg/mL gentamicin in a 37°C humidity-controlled incubator with 5% CO_2_. The initial vial was expanded and frozen down into aliquots, which were stored in liquid nitrogen with DMSO. Cultures from individual aliquots were maintained *in vitro* for ~2 months prior to being discarded. THP-1 monocytes were differentiated into macrophages by the addition of 50 nM PMA for 48 hours prior to experiments.

### Gentamicin protection phagocytosis assays with THP-1-derived macrophages

THP-1 cells were differentiated at 5 × 10^5^/mL in 24-well plates. They were then washed with PBS, and the medium was replaced with an identical medium without antibiotics. Overnight LB cultures of bacteria of indicated strains were sub-cultured at a ratio of 1:10 into LB for 60 minutes, centrifuged at 8,000 × *g* for 2 minutes, washed twice with antibiotic-free THP-1 medium, then resuspended, and bacterial density was determined by spectrophotometry at 600 nm. OD_600_ of 1.0 was empirically determined to represent 10^9^ bacteria/mL media. After dilution, bacteria were added to THP-1 cells at a multiplicity of infection (MOI) of 10 bacteria per macrophage. Plates were incubated at 37°C for 20 minutes. Then, cells were washed once with PBS, and THP-1 medium with 10× gentamicin (500 μg/mL) was added for 15 minutes. Validation experiments confirmed that 500 μg/mL killed all strains of *P. aeruginosa* equally within 15 minutes, whereas there were no differences in colony-forming units (CFUs) of any tested strains remaining after exposure to lower concentrations of 1 or 5 μg/mL.

Cells were then washed twice with PBS at 4°C, followed by lysis with 250 µL of 0.1% Triton X-100. Lysates were diluted 1:3 in PBS, and then 10 µL was plated overnight onto LB agar plates for CFU determination. Colonies were counted the next day, and bacteria per 10^6^ macrophages were calculated. MOI was also determined empirically from the stock bacterial solutions for each assay as an internal control. The average of at least three technical replicates per assay was then log-transformed, and results were combined from at least five assays performed on separate days (*n* for each experiment listed in figure legends).

### Phagocytosis competition assay

PA14 ∆*lasR* with a *lacZ* reporter gene integrated at a neutral site on the chromosome was used along with PA14 wild type. The assay was identical to above, except fixed percentages of PA14 WT and the ∆*lasR* strain were used at 100/0, 90/10, 70/30, 50/50, 30/70, 10/90, and 0/100, such that the total MOI of both strains together was constant at 10. Lysates were plated onto LB agar plates with 5-bromo-4-chloro-3-indolyl-β-D-galactoside (X-gal) for blue-white colony discrimination. All 100/0 samples were entirely white, and 0/100 samples were entirely blue, confirming the fidelity of the reporter. Total PA14 WT and ∆*lasR* colonies were counted for each well. Each experiment was performed in triplicate, and the means of technical replicates for each assay were log-transformed and combined.

### Human subjects

Subjects were recruited from our CF clinic population if they were 18–65 years old, F508del homozygous or heterozygous, at clinical baseline without symptoms of exacerbation, and had normal hemoglobin levels. Non-CF control subjects were recruited by IRB-approved flyers; smokers or those with respiratory disease, immunologic disorders, or pulmonary medication use were excluded. After informed consent was obtained and documented by our research nurse, phlebotomy was performed (100 mL whole blood isolated) for monocyte isolation and taken directly to the laboratory for downstream processing.

### Monocyte-derived macrophage generation and phagocytosis

MDMs were generated according to previously published protocols (37). Briefly, monocytes were isolated from peripheral blood by Ficoll gradient separation. They were quantified and plated directly onto plates as per the intended assay to avoid the need for detachment and re-plating. MDMs were generated by treatment with 100 ng/mL M-CSF for 7 days in RPMI/10% FCS with 50 μg/mL gentamicin. Medium was exchanged every 2–3 days. After 7 days of differentiation, MDMs were cultured in RPMI/10% FCS + gentamicin, and CFTR modulators were added for 48 hours prior to the experiment. CFTR modulators were replaced with all media exchanges. Gentamicin protection assay was performed as above. Three to four technical replicates per subject were averaged and log-transformed. Data on paired *lasR* mutants were combined with previously published data on *lasR*-WT strains (37).

### Seahorse extracellular flux assay

Monocytes were plated initially onto a Seahorse 96-well plate at 50,000 per well and differentiated into MDM directly in the wells for 7 days as above. At that point, the differentiation medium was removed, and CFTR modulators were added for 48 hours. Pa-conditioned medium was prepared in advance by culturing bacteria overnight in Seahorse mitostress assay medium, normalizing cultures to an OD_600_ of 0.500, centrifuging to pellet bacteria, then passing the supernatant through a 0.22 µm filter. This was defined as 100% conditioned medium. Aliquots were then frozen at −20°C until use.

On the day of assay, cell culture medium was exchanged for mitostress assay medium as per the manufacturer’s instructions. A mitostress assay with acute injection was run on an Agilent Seahorse XFe96 platform, with 20 µL of conditioned medium in injection port A (final concentration 10%). Plain mitostress medium was included for control wells. Oligomycin, FCCP, and rotenone/antimycin A were placed into ports B–D, respectively. Four to eight technical replicates per condition were run. Parameters, including response to injection, maximal respiration, and others, were calculated as per the manufacturer’s instructions. Data were combined with previously published results with a *lasR*-WT strain only (37).

### THP-1 Seahorse extracellular flux assay

THP-1 macrophages were differentiated directly on a Seahorse 96-well plate with 50 nM PMA at 50,000 cells/well for 48 hours. The Seahorse extracellular flux assay was then run with varying concentrations of 3OC12HSL or C4HSL in injection port A. Five to six technical replicates were run per condition for each assay and averaged. Five independent experiments were run.

### Cytokine analysis

MDMs were differentiated for 7 days as above and then treated for 48 hours with CFTR modulators or DMSO. Bacteria were prepared in an identical manner to the phagocytosis assays. After exchange for antibiotic-free media, MDMs were infected with indicated strains at an MOI of 10 for 2 hours, then washed and placed in media with gentamicin for an additional 2 hours. Supernatants were then collected, centrifuged at 14,000 × *g* at 4°C for 10 minutes, transferred to a new tube, and frozen at −20°C until needed. Samples were batched, and cytokine multiplex analysis was run by the Dartmouth Immunology and Flow Cytometry Core. One replicate per condition was run. Samples with levels below the limit of the detection of the assay were set to a concentration of 0, and data were log-transformed prior to analysis.

### Figure generation and statistical analysis

Figures were generated with GraphPad Prism 10. Statistical analyses were performed separately in R, with mixed model linear analysis using the nlme package version 3.1. Models include all variables of interest as fixed variables and assay date (for THP-1 experiments) or subject (for MDM experiments) as random variables.

## SUPPLEMENTAL MATERIAL

Data set S1 msphere.00702-25-s0001.xlsx Annotation of genetic differences between DH2417 and DH2415.

Supplemental figures msphere.00702-25-s0002.pdf Figures S1-S8.

Supplemental methods msphere.00702-25-s0003.docx Methods for Fig. S1.

ASM does not own the copyrights to Supplemental Material that may be linked to, or accessed through, an article. The authors have granted ASM a non-exclusive, world-wide license to publish the Supplemental Material files. Please contact the corresponding author directly for reuse.
