# Erratum for Aridgides et al., “*Pseudomonas aeruginosa lasR* mutants resist phagocytosis and alter inflammatory cytokine production by cystic fibrosis macrophages”

**DOI:** 10.1128/msphere.00430-26

**Published:** 2026-07-10

**Authors:** Daniel S. Aridgides, Diane L. Mellinger, Lorraine L. Gwilt, Anthony R. Correa, Carson E. Finger, Jay Goddard, Thomas H. Hampton, Dallas L. Mould, Deborah A. Hogan, Alix Ashare

## ERRATUM

Volume 11, no. 4, e00702-25, 2026, https://doi.org/10.1128/msphere.00702-25. Byline: Anthony R. Correa’s name should appear as shown in this Erratum.

