# Supplementary material for: Pseudomonas aeruginosa lasR mutants resist phagocytosis and alter inflammatory cytokine production by cystic fibrosis macrophages
Source: mSphere. 2026 Apr 2;11(4):e00702-25. doi: 10.1128/msphere.00702-25 (PMC13123724; doi:10.1128/msphere.00702-25)
Supplement: Supplemental figures — Figures S1-S8. [file msphere.00702-25-s0002.pdf]

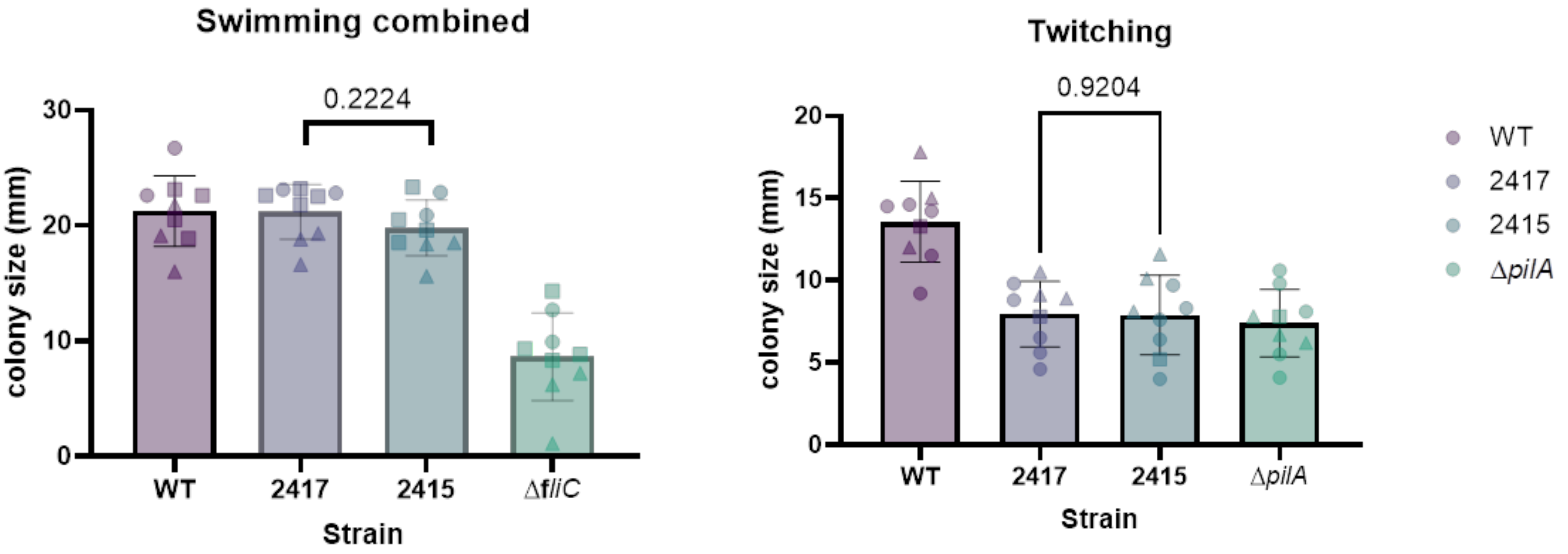

Figure S1. *lasR* mutation does not impact *P. aeruginosa motility.* A) PA14WT, DH2417, DH2415 motility were measured with a swimming assay on 0.3% agar plates (n=3 independent assays, three technical replicates each). ΔfliC which carries a mutation in type A flagellin was used as a negative control for swimming motility. p=0.2254 for DH2415 vs DH2417 by paired t-test of means from each assay. **B)** Twitching motility was measured in the indicated strains by inoculating the middle of a 1.5% agar plate followed by incubation for 16 hours then staining with crystal violet. Colony diameter was measured as a readout for twitching motility. ΔpilA carries a mutation in a major component of the type IV pilus and served as a negative control for twitching motility (n=3 independent assays with three technical replicates each). p= 0.9204 for DH2417 vs DH2415 by paired t-test of means from each assay.

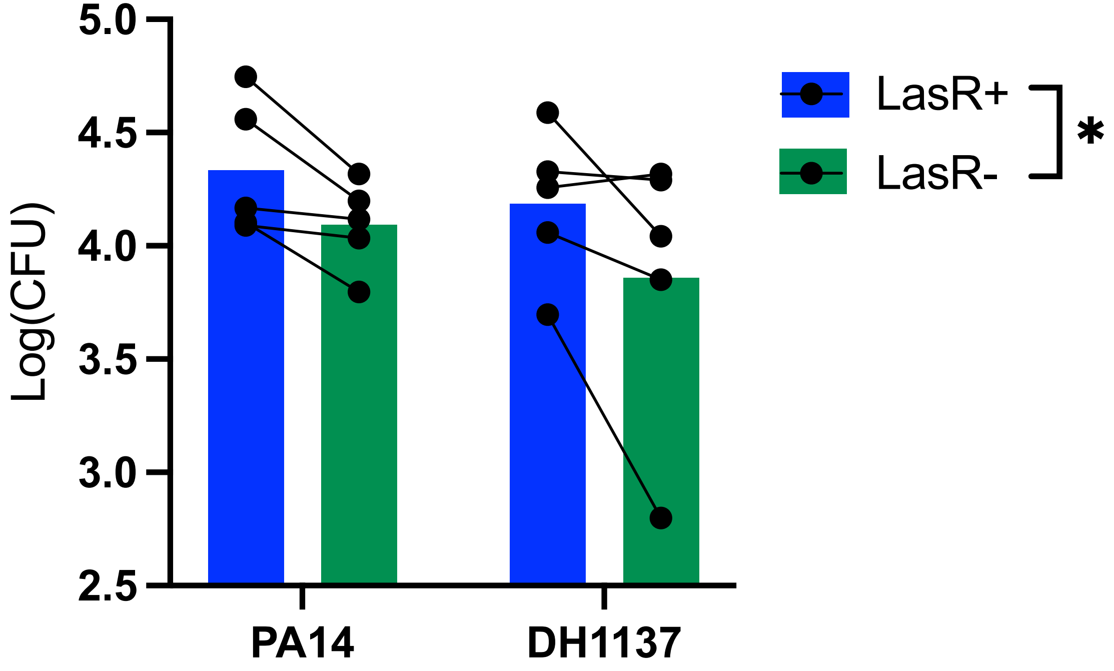

Figure S2. LasR mutation confers resistance to opsonic phagocytosis. Phagocytosis assay was performed with THP-1 derived macrophages as in **Figure 1**, with exception that 10% human serum was added to the bacteria prior to phagocytosis and maintained throughout the engulfment step. * $p<0.05$ for LasR mutation affecting phagocytosis globally. Individual comparisons on the PA14 and DH1137 background were not statistically significant.

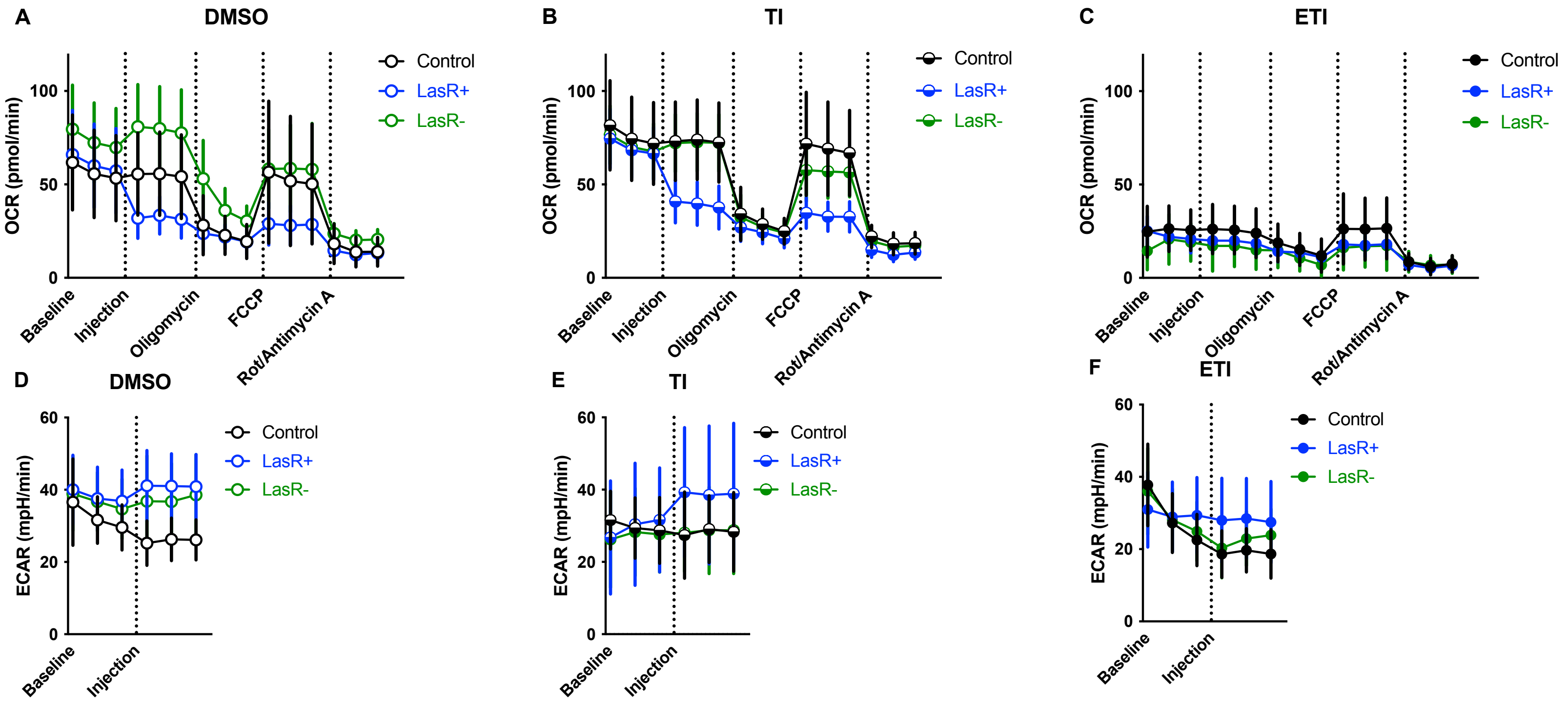

Figure S3. Effects of conditioned medium and CFTR modulators on CF MDM metabolism.
A-C) Representative oxygen consumption rate tracings from a CF subject MDMs pretreated with DMSO, TI, or ETI. **D-F**) Representative extracellular acidification rates from the same subject.

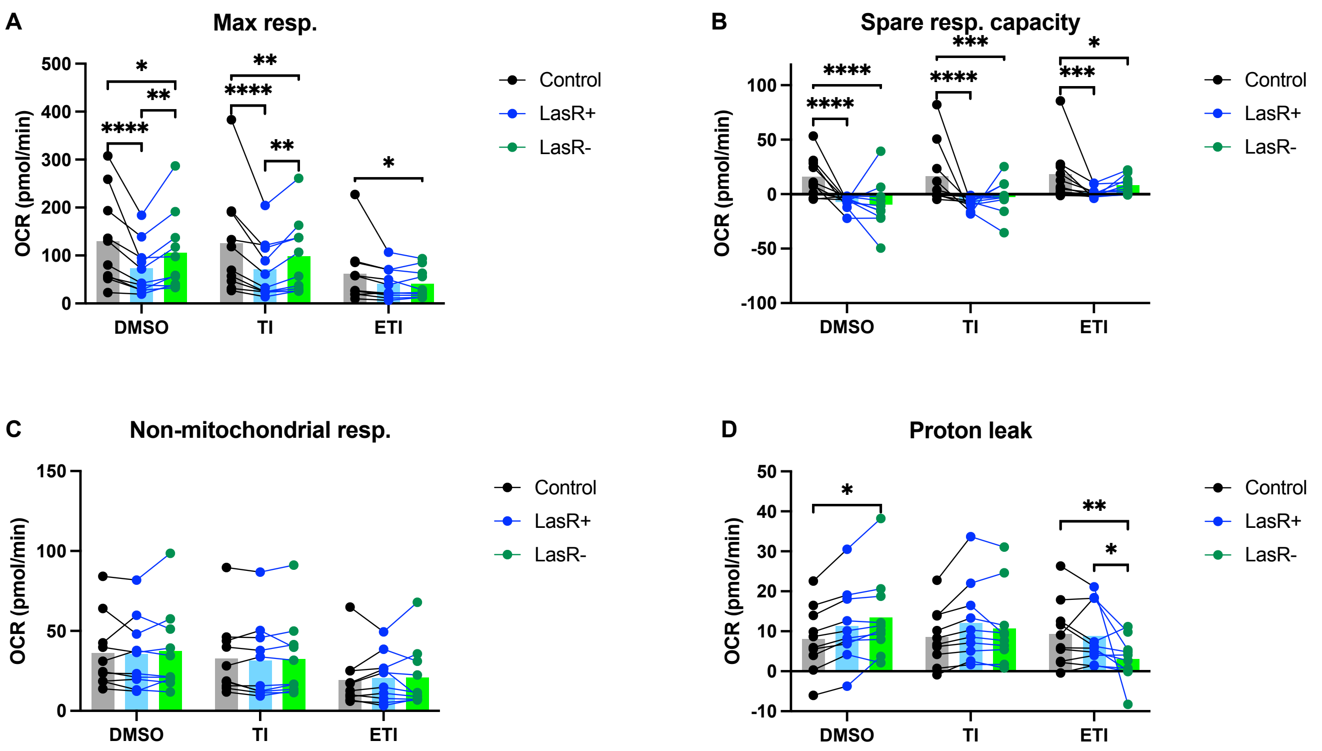

Figure S4. Additional Seahorse parameters from CF MDMs. Calculated parameters from the Seahorse assay in **Figure 5A-F** are displayed. *p<0.05, **p<0.01, ***p<0.001, ****p<0.0001 by mixed model linear regression for indicated comparisons.

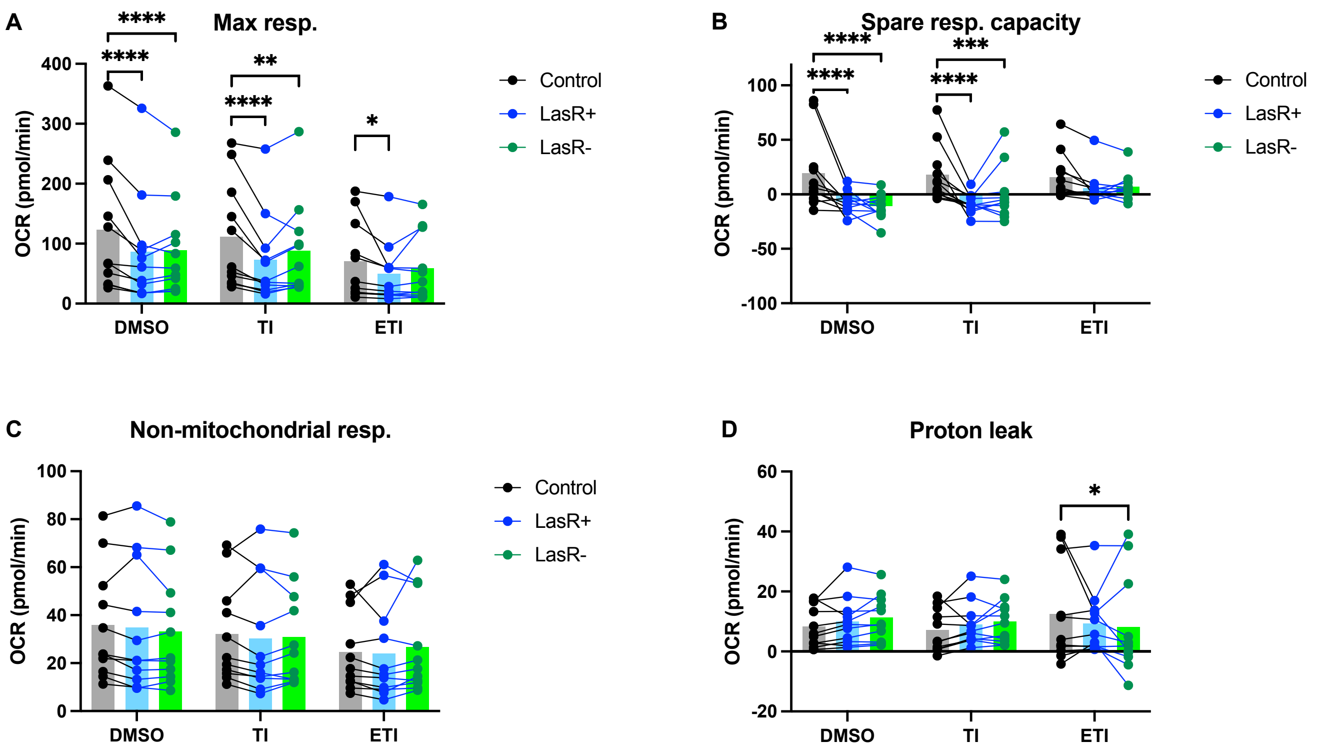

Figure S5. Additional Seahorse parameters from nonCF MDMs. Calculated parameters from the Seahorse assay in ∫ are displayed. *p<0.05, **p<0.01, ***p<0.001, ****p<0.0001 by mixed model linear regression for indicated comparisons.

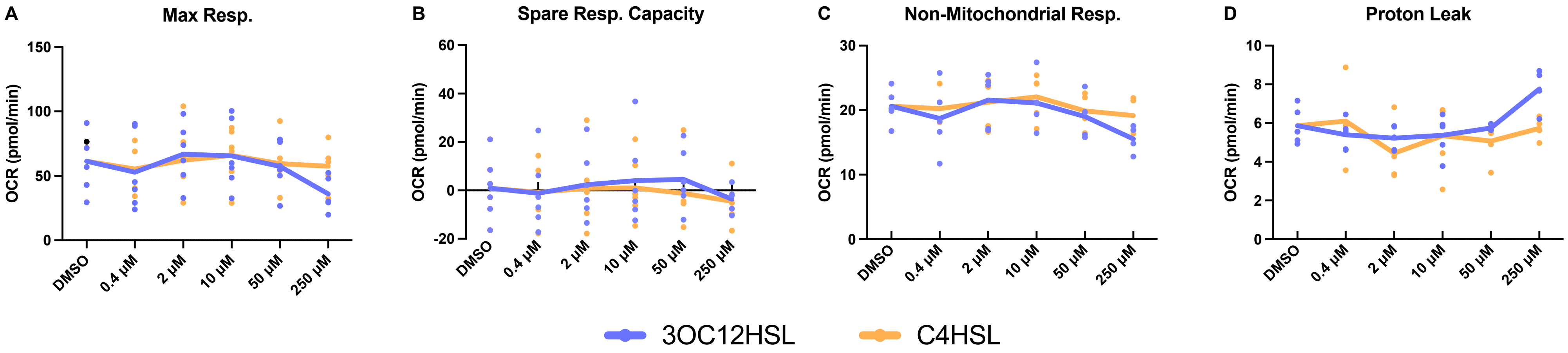

Figure S6. Additional Seahorse parameters from THP-1s with acute injection of HSLs. Calculated parameters from the Seahorse assay in **Figure 6** are displayed. Points represent the means of technical replicates from individual experiments, with thick lines at the means.

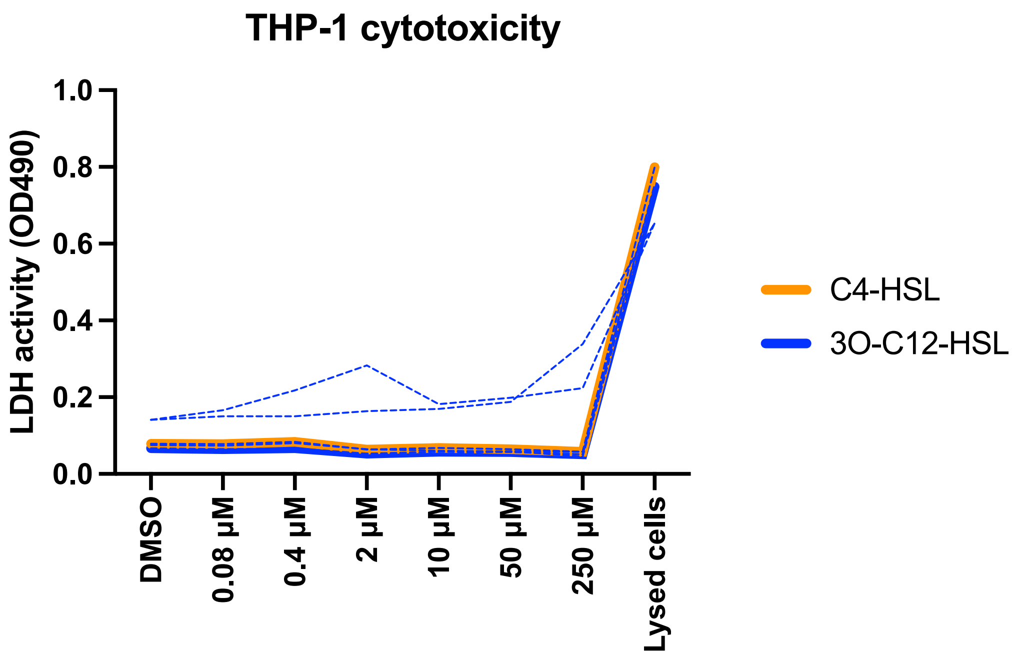

Figure S7. Homoserine lactones are not directly cytotoxic to THP-1 macrophages under measured conditions. THP-1 cells were plated onto 24-well plates with 50 nM PMA for 48 hours, washed with PBS, then treated with indicated doses of HSL for one hour in fresh THP-1 medium. Supernatants were collected and an LDH assay was run according to manufacturer's instructions. Dashed lines represent the means of three technical replicates each, during three independent experiments, with thick lines at the means of the three experiments. Cells incubated with supplied lysis buffer were used as a positive control for total LDH activity.

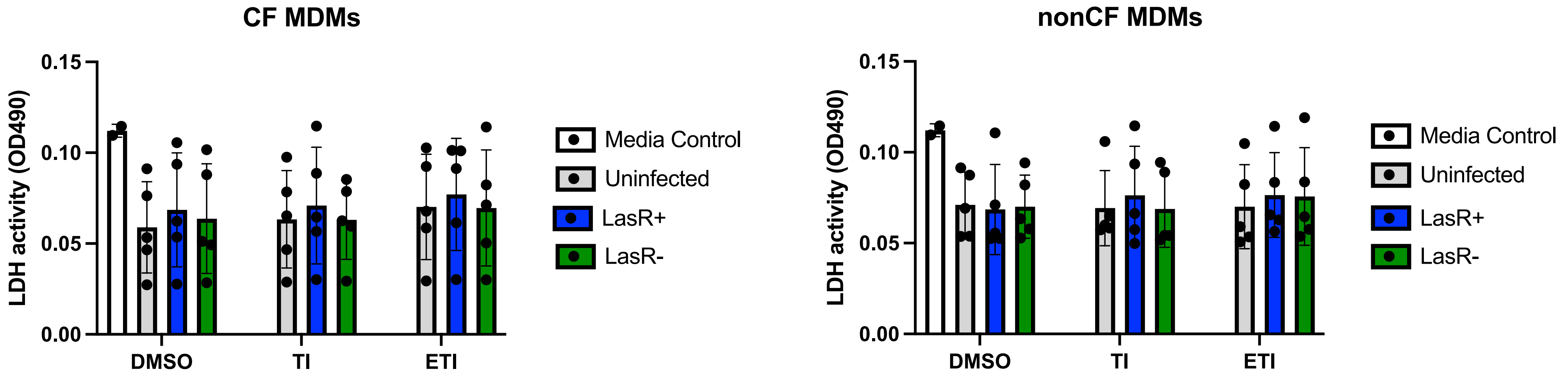

Figure S8. MDM cytotoxicity is minimal after CFTR modulator treatment with or without *P. aeruginosa* infection. CF or nonCF (n=5 each) MDMs were pre-treated with CFTR modulators for 48 hours, cells were washed, placed in antibiotic-free medium, CFTR modulators were replaced, and then cells were infected for 2 hours with DH1137 (LasR+) or DH1136 (LasR-) clinical strains of *P. aeruginosa*. Uninfected control wells were run in parallel. Supernatants were collected after 2 hours and frozen at -20° C until needed. LDH assay was run per manufacturer's instructions. Media control (RPMI with 10% FBS and gentamicin) was run as a control. There were no statistically significant differences between CFTR modulator treatments or infection in either cohort.
