# Supplementary material for: Pseudomonas aeruginosa lasR mutants resist phagocytosis and alter inflammatory cytokine production by cystic fibrosis macrophages
Source: mSphere. 2026 Apr 2;11(4):e00702-25. doi: 10.1128/msphere.00702-25 (PMC13123724; doi:10.1128/msphere.00702-25)
Supplement: Supplemental methods — Methods for Fig. S1. [file msphere.00702-25-s0003.docx]

**Supplemental Methods**

**Opsonic phagocytosis assay**

THP-1 cells were differentiated into macrophages with addition of 50 nM PMA as done previously. Phagocytosis assay was carried out identically to **Figure 1A and 1B** however 10% serum was added to bacterial cultures during the subculture phase, as well as to the culture medium during uptake to allow complement to adhere to bacteria prior to uptake. Pooled serum from 4 independent donors was used from banked frozen samples acquired during blood draws for MDM generation. Experiment was performed with 2-4 technical replicates per condition in 5 separate assays. Means of technical replicates from each assay were analyzed.

**Swimming assay.**

**Swim Medium** - To make 500 mL agar: 382 mL of water, 1.5 g of agar (final concentration: 0.3%), 100 mL of filter sterilized 5x M63 salt solution, 5mL of 20 % glucose (final concentration: 0.2%), 12.5 mL of 20 % casamino acids (final concentration: 0.5%), 0.5mL of 1 M MgSO4 (final concentration: 1 mM MgSO4). Twenty five mL of molten medium was poured into petri plates and allowed to solidify at room temperature (RT).

**Inoculation, Incubation and Imaging -** Using a sterile toothpick, bacteria freshly grown on LB plates were collected on the tip of the toothpick, then stabbed into the agar without contacting the bottom of the plastic dish. Plates were incubated upright at 37 °C for 16 h then imaged. Three technical replicates per strain were used in three independent assays.

**Twitching assay**

**Medium—**Twitching medium contains 1.5% agar, and 10 g of tryptone, and 5 g of NaCl per liter. Each plate contained 25 ml of agar.

**Twitching assay -** Using a sterile toothpick, bacteria from LB plates were stabbed into the agar layer of the plate making sure to touch the bottom of the plastic dish. Plates were incubated at 37 °C for 16 h. Following incubation and 200 µL pipette tip was used to separate the agar for the edge of plastic dish and then to fully remove the agar out of the bottom. 500 µL of 0.1% crystal violet stain was pipetted on top of the locations where the bacteria was inoculated and stained for 10 min. After 10 min the plates were rinsed with water to remove excess stain and image. Three technical replicates per strain were used in three independent assays
